# Exercise intolerance in heart failure with preserved ejection fraction: Causes, consequences and the journey towards a cure

**DOI:** 10.1113/EP090674

**Published:** 2023-12-08

**Authors:** Kanokwan Bunsawat, Michael D. Nelson, Christopher M. Hearon, D. Walter Wray

**Affiliations:** ^1^ Geriatric Research, Education, and Clinical Center, George E. Wahlen Department of Veterans Affairs Medical Center Salt Lake City Utah USA; ^2^ Department of Internal Medicine, Division of Geriatrics University of Utah Salt Lake City Utah USA; ^3^ Department of Kinesiology University of Texas at Arlington Arlington Texas USA; ^4^ Department of Applied Clinical Research The University of Texas Southwestern Medical Center Dallas Texas USA; ^5^ Department of Nutrition and Integrative Physiology University of Utah Salt Lake City Utah USA

**Keywords:** blood flow, exercise training, heart failure, vascular physiology

## Abstract

Heart failure with preserved ejection fraction (HFpEF) accounts for over 50% of all heart failure cases nationwide and continues to rise in its prevalence. The complex, multi‐organ involvement of the HFpEF clinical syndrome requires clinicians and investigators to adopt an integrative approach that considers the contribution of both cardiac and non‐cardiac function to HFpEF pathophysiology. Thus, this symposium review outlines the key points from presentations covering the contributions of disease‐related changes in cardiac function, arterial stiffness, peripheral vascular function, and oxygen delivery and utilization to exercise tolerance in patients with HFpEF. While many aspects of HFpEF pathophysiology remain poorly understood, there is accumulating evidence for a decline in vascular health in this patient group that may be remediable through pharmacological and lifestyle interventions and could improve outcomes and clinical status in this ever‐growing patient population.

## Abstract (highlights)

- **What is the topic of this review?**
  This symposium review provides an integrative view of how disease‐related changes in cardiac, vascular and skeletal muscle function contribute to exercise intolerance in patients with heart failure with preserved ejection fraction (HFpEF).
- **What advances does it highlight?**
  Emerging evidence continues to highlight the importance of considering both cardiac and non‐cardiac abnormalities in patients with HFpEF, with accumulating evidence for ‘plasticity’ in vascular function that emphasizes the need for a greater focus on strategies targeting the peripheral circulation as a means to improve exercise intolerance.

## 1 INTRODUCTION

**FIGURE 1:**
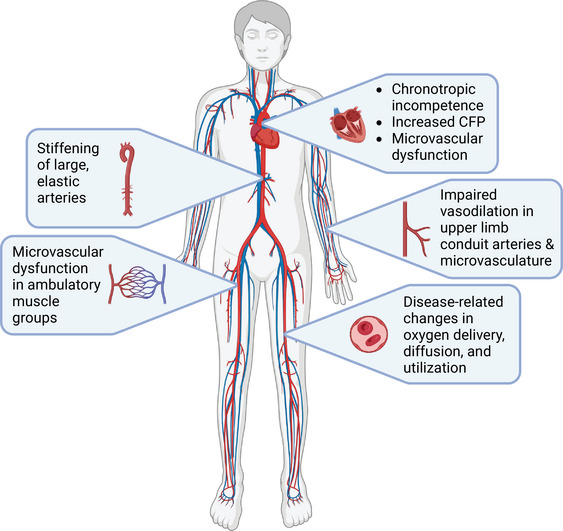
Overview of disease‐related changes in cardiac, vascular and skeletal muscle function that contribute to exercise intolerance in patients with HFpEF. CFP, cardiac filling pressure. Created with Biorender.com.

Heart failure with preserved ejection fraction (HFpEF) accounts for over 50% of all heart failure cases in the United States and continues to rise (Tsao et al., 2018). This clinical syndrome is associated with poor quality of life and a dismal prognosis, with a 5‐year mortality rate that ranges from 50% to 75% (Dunlay et al., 2017). Dyspnoea upon exertion and severe exercise intolerance are defining features of HFpEF, symptoms that derive from disease‐related changes in multiple organ systems (Nayor et al., 2020). Indeed, beyond disease‐related changes in cardiac function such as elevated atrial filling pressure (Borlaug et al., 2010; Pfeffer et al., 2019; Reddy et al., 2018; Samuel et al., 2021) and chronotropic incompetence (Borlaug et al., 2010, Borlaug, Kane et al., 2016; Haykowsky et al., 2011), there is emerging evidence for abnormalities in vascular, skeletal muscle and pulmonary function that each appear to contribute to exercise intolerance in this patient group (Nayor et al., 2020) (Figure 1). The heterogeneous involvement of organ systems likely stems from the ‘constellation of comorbidities’ that characterize the HFpEF phenotype, including hypertension, pulmonary disease, type‐2 diabetes mellitus, morbid obesity and physical inactivity (Deichl et al., 2022). Facing the combined effect of multi‐organ involvement and polymorbidity, the search for effective treatment of HFpEF has yielded largely negative results. Indeed, while a small number of studies have identified the efficacy of pharmacological and lifestyle interventions on select physiological parameters, the majority of randomized clinical trials have been largely ineffective in identifying strategies for improving hard clinical outcomes in patients with HFpEF (Redfield & Borlaug, 2023). Thus, the complexity of this unique patient group continues to pose significant challenges in determining both underlying pathophysiology and in optimizing clinical care of patients with HFpEF.

With recognition of the need to adopt an integrative approach when investigating HFpEF, the symposium ‘Exercise Intolerance in Heart Failure with a Preserved Ejection Fraction (HFpEF): Causes, Consequences, and the Journey Towards a Cure’, which took place at the ACSM 2022 World Congress on the Basic Science of Exercise and Vascular Health, brought together experts in several areas of physiology to explore how disease‐related changes in cardiac, vascular, and skeletal muscle function contribute to exercise intolerance in HFpEF. Select studies utilizing unique therapeutic approaches for mitigating vascular dysregulation in this clinical population were also presented, with an overall focus on new strategies for improving physical function and exercise capacity. Highlighted below are the salient points from each presentation, which together provided a comprehensive and contemporary view of ongoing efforts in further establishing the causes and consequences of exercise intolerance in patients with HFpEF, as well as an update on the journey towards improving outcomes in this ever‐growing patient group.

## 2 CARDIAC FUNCTION IN HFpEF

Abnormalities in cardiac structure, function and reserve each play a pivotal role in the pathophysiology of exercise intolerance in HFpEF (Pfeffer et al., 2019). Chronotropic incompetence and impairments in stroke volume reserve both limit the rise in cardiac output (CO) during exercise in HFpEF (Borlaug et al., 2010, Borlaug, Kane et al., 2016; Haykowsky et al., 2011), the latter of which is largely explained by abnormalities in systolic function (Borlaug et al., 2010; Haykowsky et al., 2011; Pandey et al., 2018) related to both contractile dysfunction and cardiac afterload (Borlaug et al., 2010; Reddy et al., 2017). Emerging evidence also implicates left atrial (LA) dysfunction as an important pathophysiological mechanism driving exercise intolerance in HFpEF, with impaired LA function and increased LA stiffness associated with abnormal exercise haemodynamics and peak oxygen uptake (Freed et al., 2016; Kusunose et al., 2012; Singleton et al., 2022). Similar observations have also been made with right atrial function and reserve (Kagami et al., 2022).

In addition to limitations in cardiac reserve, impairments in left ventricular diastolic compliance, relaxation reserve, and pericardial restraint lead to a marked rise in pulmonary capillary wedge pressure (PCWP) during exercise in HFpEF (Borlaug et al., 2010; Pfeffer et al., 2019; Reddy et al., 2018; Samuel et al., 2021). However, whether such steep elevations in cardiac filling pressures independently limits exercise remains incompletely understood, with at least one recent report dissociating the rise in PCWP from exercise tolerance (Sarma et al., 2022). Increases in PCWP during exercise, however, may cause lung congestion that could acutely promote dyspnoea (Obokata et al., 2018; Reddy et al., 2019). For example, using semi‐quantitative lung ultrasound, Simonovic et al. (2021) found that submaximal exercise increased the extent of B‐line formation (a marker of extravascular lung water) in HFpEF, coincident with significant variations in natriuretic peptides, greater tricuspid regurgitation, and diastolic dysfunction (i.e., *E*/*e*′). Likewise, Burrage et al. (2021) found a significant increase in lung water content in HFpEF following submaximal exercise (20 W), measured using a novel pulmonary proton density magnetic resonance imaging sequence. Moreover, using computed tomography, an increase in lung congestion immediately following submaximal exercise in HFpEF has been reported that appears to coincide with measurable impairments in lung diffusion capacity (Fermoyle et al., 2020, 2021). Thus, with recognition that the mechanisms responsible for exercise intolerance in HFpEF extend beyond the derangements in cardiac function that characterize this patient group, the contribution of abnormalities in cardiac structure, function and reserve should not be overlooked.

## 3 CORONARY MICROVASCULAR FUNCTION IN HFpEF

In patients with HFpEF, the presence of endothelium‐dependent and ‐independent coronary microvascular dysfunction is documented by reduced coronary blood flow response to intracoronary infusion of acetylcholine using Doppler flow wire with quantitative angiography (Yang et al., 2020) and an attenuated hyperaemic increase in coronary flow reserve in response to adenosine administration (Arnold et al., 2022; Yang et al., 2020). Similar to the peripheral vasculature, coronary microvascular endothelial dysfunction is thought to be mediated, in part, by oxidative stress and reduced nitric oxide (NO) bioavailability (Franssen et al., 2016). Interestingly, while there is a lack of relationship between endothelium‐dependent microvascular function and cardiac haemodynamics (Yang et al., 2020), endothelium‐independent coronary microvascular function has been associated with long‐term clinical outcomes (Arnold et al., 2022), mortality risks (Yang et al., 2020), and exertional haemodynamic abnormalities (i.e., higher filling pressure at peak exercise) and exercise intolerance in patients with HFpEF (Ahmad et al., 2021; Mahfouz et al., 2020). Therefore, given that patients with HFpEF may present with endothelial dysfunction at both the conduit artery and microvascular levels (discussed below), identification of strategies to improve this aspect of HFpEF‐related pathophysiology is of clinical significance.

## 4 ARTERIAL STIFFNESS AND AORTIC HAEMODYNAMICS IN HFpEF

Large elastic arteries, particularly the proximal aorta, play an important role in buffering oscillations in arterial blood pressure and blood flow that occur in response to LV contraction (Schultz et al., 2013). Age‐ and disease‐associated large artery stiffening and increased peripheral resistance decrease the buffering capacity of the proximal aorta, resulting in a greater mid‐late systolic afterload (augmentation pressure) and pulse pressure for any given stroke volume (Baksi et al., 2009). Patients with HFpEF have marked arterial stiffening beyond that observed with ageing (Hundley et al., 2001) and hypertension (Desai et al., 2009), or heart failure with a reduced ejection fraction (HFrEF) (Balmain et al., 2007). In patients with HFpEF, elevations in markers of arterial stiffness and associated wave reflections are associated with impaired systolic and diastolic function at rest and adverse cardiovascular outcomes (Namasivayam et al., 2022). Interestingly, while resting measures of arterial stiffness are poor independent predictors of functional capacity in HFpEF (Hundley et al., 2001; Kitzman, Herrington et al., 2013; Tartière‐Kesri et al., 2012; Zern et al., 2021), exercise unmasks a pathophysiological interaction between arterial stiffness, stroke volume and peripheral resistance (Reddy et al., 2017; Tartière‐Kesri et al., 2012) that contributes to greater wave reflection, augmentation pressure, pulse pressure and mid/late‐systolic LV afterload in HFpEF (Reddy et al., 2017; Tartière‐Kesri et al., 2012; Zern et al., 2021). The resulting increase in cardiac afterload is associated with diminished myocardial performance (Chirinos, Segers et al., 2019; Reddy et al., 2017), greater PCWP/CO response to exercise, and lower peak ${\dot{V}}_{O_{2}}$ (Zern et al., 2021). However, the extent to which central arterial stiffness and pressure amplification contribute to exercise intolerance may be considerably heterogeneous due to the presence of specific comorbidities (Chirinos, Bhattacharya et al., 2019; Jain et al., 2021) and biological sex (Lau et al., 2022), and thus additional studies are needed to define the contribution of arterial stiffness to specific HFpEF phenotypes.

Several studies have investigated the effect of lifestyle and pharmacological interventions on central arterial stiffness and haemodynamics in patients with HFpEF. Moderate‐intensity aerobic exercise performed for 16–20 weeks showed no improvement in several indices of resting vascular stiffness/compliance or pulse wave velocity (PWV) in elderly patients with HFpEF (Kitzman, Brubaker et al., 2013, 2016). Similarly, neither longer duration (1 year) progressive endurance exercise training (Fujimoto et al., 2012) nor high intensity interval training (Gevaert et al., 2023) improved markers of vascular stiffness in patients with HFpEF. Interestingly, while caloric restriction leading to significant weight loss was insufficient to change arterial pulse wave velocity in older obese patients with HFpEF (Kitzman et al., 2016), the combination of aerobic exercise training and caloric restriction, with the addition of resistance exercise training, was effective in reducing arterial PWV in patients with HFpEF (Brubaker et al., 2022). While this combination of lifestyle interventions suggest that arterial stiffness is a modifiable feature of HFpEF pathophysiology, further investigations are needed to develop additional lifestyle interventions capable of reversing arterial stiffness in HFpEF, and to identify strategies capable of mitigating arterial stiffness earlier in the progression of HFpEF (Hearon, Dias et al., 2022).

The most commonly investigated pharmacological interventions to improve central aortic haemodynamics target the nitrate–nitrite–NO pathway (Chirinos et al., 2017). Inorganic nitrite administration reduces the magnitude of central aortic wave reflections in patients with HFpEF (Chirinos et al., 2017; Zamani et al., 2015), but has modest (Zamani et al., 2015) or no effect (Borlaug et al., 2018) on peak ${\dot{V}}_{O_{2}}$ and exercise capacity. However, inorganic nitrite may have benefits during submaximal exercise (Borlaug, Melenovsky et al., 2016; Reddy et al., 2017, 2021), including improved oxygen uptake kinetics, cardiac output and lower pressure augmentation profile likely due to combined effects of nitrite on pulmonary, cardiac and peripheral vasodilatory function (Reddy et al., 2021). Future studies should consider the differential contribution of vascular stiffness within specific phenogroups of HFpEF which may dictate the responsiveness of interventions designed to target vascular stiffness.

## 5 PERIPHERAL CONDUIT VESSEL FUNCTION IN HFpEF

Conduit artery endothelial dysfunction, assessed via brachial artery flow‐mediated dilatation (FMD) testing, independently predicts cardiovascular disease‐related morbidity and mortality (Yeboah et al., 2009). The vasodilatory response to brachial artery FMD testing is partly mediated by NO (Green et al., 2014), suggesting that FMD may serve as a bioassay for NO bioavailability. Given the continued interest in pharmacotherapies targeting the NO pathway in patients with HFpEF, recent studies have sought to determine the magnitude of endothelial dysfunction, and whether it could be restored, in this patient group. While initial work failed to identify differences in FMD between patients and age‐matched controls in the upper (Gevaert et al., 2023; Haykowsky, Herrington et al., 2013) or lower (Hundley et al., 2007) limbs, more recent work has reported lower FMD values in HFpEF compared to healthy age‐matched (Kishimoto et al., 2017) and hypertensive (Farrero et al., 2014; Marechaux et al., 2016) control subjects. Interestingly, Lee, Barrett‐O'Keefe, Garten et al. (2016) observed that a decrease in %FMD in HFpEF compared to healthy age‐matched controls was no longer evident after the FMD response was normalized for the shear stimulus. While these disparate findings regarding endothelium‐dependent dilatation are most likely attributable to the marked heterogeneity within this patient population, collectively, the majority of studies to date indicate some decrement in conduit artery endothelial function in patients with HFpEF (Ambrosino et al., 2021).

Every 1% change in FMD has been reported to confer a reciprocal ∼13% change in risk for cardiovascular disease events (Inaba et al., 2010), suggesting that strategies seeking to improve endothelial function could provide a significant benefit in patients with HFpEF. Interestingly, neither moderate‐intensity endurance (Kitzman, Brubaker et al., 2013) nor high‐intensity interval (Angadi et al., 2015) exercise training improved FMD in patients with HFpEF, though some caution is warranted in interpretation, as training‐induced changes in vessel diameter and shear stimulus were not evaluated. In contrast, nutraceutical and pharmacological interventions have proven more effective. Both acute antioxidant supplementation (Ratchford et al., 2019) and 7 days of l‐citrulline administration (Ratchford et al., 2023) improved FMD in patients with HFpEF. While there is emerging evidence for favourable effects of newly developed heart failure pharmacotherapies such as sacubitril–valsartan on conduit artery endothelial function in HFrEF (Bunsawat, Ratchford, Alpenglow, Park et al., 2021), the impact of this drug class on vascular outcomes has not been determined in patients with HFpEF. However, expanded use of sacubitril–valsartan and other therapeutics (e.g., soluble guanylate cyclase stimulators and sodium–glucose cotransporter 2 inhibitors) in patients with EF > 40% presents an opportunity to determine the effect of these drugs on vascular health in HFpEF.

## 6 PERIPHERAL MICROVASCULAR FUNCTION IN HFpEF

Accumulating evidence has demonstrated a disease‐related attenuation in microvascular function in the peripheral circulation of patients with HFpEF, as evidenced by a marked reduction in the reactive hyperaemic response following lower arm cuff occlusion compared to age‐ and sex‐matched control participants (Lee, Barrett‐O'Keefe, Garten et al., 2016) and similarly aged hypertensive control participants (Marechaux et al., 2016). Locomotor muscle microvascular dysfunction, as determined by passive limb movement testing, has also been reported in patients with HFpEF (Francisco et al., 2021). Responses to passive limb movement are predominantly NO‐mediated, suggesting that disease‐related changes in NO signalling could be present, and may contribute to functional limitations (Trinity et al., 2012). Although a recent pilot study seeking to improve NO bioavailability through l‐Cit administration produced modest improvements in lower limb microvascular reactivity in patients with HFpEF, whether peripheral microvascular dysfunction can be mitigated through lifestyle or pharmacological interventions remains a largely unexplored area of investigation (Ratchford et al., 2023).

## 7 PERIPHERAL DETERMINANTS OF OXYGEN UTILIZATION IN HFpEF

During graded cardiopulmonary exercise testing, functional capacity (peak ${\dot{V}}_{O_{2}}$) can be split into its components according to the Fick principle to quantify the relative contribution of central (${\dot{Q}}_{C}$) and peripheral (a–vO_2_ difference) components of exercise intolerance. While lower peak ${\dot{Q}}_{C}$ observed during exercise in HFpEF can be interpreted as a central limitation, cardiac output responses during exercise should always be interpreted in the context of metabolic demand (Bhella et al., 2011). When the ${\dot{Q}}_{C}$ response to exercise is normalized to ${\dot{V}}_{O_{2}}$ (${\dot{Q}}_{C}$/${\dot{V}}_{O_{2}}$ slope) in patients with HFpEF, most studies report a preserved or exaggerated ${\dot{Q}}_{C}$/${\dot{V}}_{O_{2}}$ relationship (Bhella et al., 2011; Haykowsky et al., 2011; Namasivayam et al., 2022; Obokata et al., 2017, 2018; Zamani et al., 2020). While these findings are not universal (Abudiab et al., 2013) and are likely to be influenced by body position (supine vs. upright) and comorbidities (Obokata et al., 2017), they do support several investigations indicating that impaired oxygen extraction (a–vO_2_ difference) is a key determinant of exercise intolerance in HFpEF (Bhella et al., 2011; Dhakal et al., 2015; Haykowsky et al., 2011). Broadly, a–vO_2_ difference is determined by convective transport of oxygen via blood flow, the diffusive transport of oxygen from red blood cells to the mitochondria, and the utilization of oxygen within the myocyte. HFpEF patients appear to exhibit abnormalities in each of these steps along the oxygen transport cascade, as discussed below.

## 8 EXERCISING SKELETAL MUSCLE BLOOD FLOW IN HFpEF

Patients with HFpEF consistently demonstrate a blunted fall in systemic vascular resistance during exercise, especially in the upright position, which is a primary contributor to exercise intolerance (Pandey et al., 2018). Investigations utilizing smaller muscle mass exercise that isolate peripheral mechanisms of oxygen transport and utilization independent of central cardiopulmonary limitations have identified striking impairments in the regulation of skeletal muscle blood flow in patients with HFpEF. Indeed, studies utilizing isolated knee‐extensor exercise (up to 15 W, ∼75–90% of peak work rate) indicate that patients with HFpEF have 15–25% lower leg blood flow compared to age‐matched control participants due primarily to a lower vasodilatory response to exercise (Hearon, Samels et al., 2022; Lee, Barrett‐O'Keefe, Nelson et al., 2016). Similarly, investigations employing handgrip exercise (30–45% of maximal voluntary contraction, MVC) documented a 20–40% lower forearm blood flow in patients with HFpEF compared to age‐matched hypertensives that was apparent primarily at higher exercise intensities (Ratchford et al., 2020). However, findings in the forearm are not universal, as another investigation indicates preserved forearm blood flow when compared to age matched and hypertensive controls at ∼70% of MVC (Zamani et al., 2020).

The mechanisms responsible for the decrement in blood flow during exercise are unclear, and may differ according to HFpEF phenotype. For example, patients with HFpEF who are obese have 30–40% lower forearm blood flow compared to those without obesity during progressive, rhythmic handgrip exercise that is accompanied by a marked elevation in proinflammatory cytokines (Ratchford et al., 2022). The degree of adiposity is also associated with poor peripheral oxygen utilization (a–vO_2_ difference) during handgrip exercise (Zamani et al., 2020). The direct mechanism for obesity‐associated impairments in peripheral oxygen utilization are likely multifactorial and could include the combined effects of adipose tissue on blood flow distribution and skeletal muscle mitochondrial function (Molina et al., 2016). Disease‐related changes in circulating vasoactive substances may also play a prominent role. Indeed, there is now accumulating evidence for sympathetic nervous system (SNS) overactivity at rest (Seravalle et al., 2019) and during exercise (Badrov et al., 2022; Bunsawat, Ratchford, Alpenglow, Ryan et al., 2021) in patients with HFpEF, which may promote excess vasoconstriction and limit the hyperaemic response during exercise. A recent study from Alpenglow et al. (2023) supports this interesting possibility, providing initial evidence for a diminished ability to attenuate SNS‐mediated vasoconstriction in the exercising limb of patients with HFpEF compared to healthy aged‐matched controls. Further, prior work has also identified an important role of non‐adrenergic vasoconstrictor signalling pathways including endothelin‐A (Barrett‐O'Keefe et al., 2015) and the renin–angiotensin–aldosterone system (Wray et al., 2008) in the regulation of exercising muscle blood flow in older, healthy adults. Remarkably, almost nothing is currently known regarding the role of non‐adrenergic signalling in the regulation of muscle blood flow during exercise in patients with HFpEF, and thus further studies are certainly warranted to explore these potential mechanisms of vascular dysregulation, and how it may relate to exercise intolerance, in this patient group.

## 9 SKELETAL MUSCLE OXYGEN UTILIZATION IN HFpEF

Patients with HFpEF exhibit increased intermuscular fat deposition and poor skeletal muscle functional performance compared to healthy age‐matched controls (Haykowsky, Brubaker et al., 2013), which are associated with reduced peak ${\dot{V}}_{O_{2}}$ (Haykowsky et al., 2014, 2018). Further, capillary‐to‐fibre ratio is reduced in HFpEF, which may impair diffusive capacity, and a shift towards greater type II muscle fibres is associated with reduced oxidative capacity and efficiency (Kitzman et al., 2014; Zamani et al., 2021) and slower ${\dot{V}}_{O_{2}}$ onset kinetics during submaximal exercise (Hearon et al., 2019; Krustrup et al., 2008). Severe structural, biochemical and bioenergetic abnormalities have been identified in HFpEF, including impaired mitochondrial content, abnormal mitochondrial fusion and reduced activity of citrate synthase and other proteins integral to proper mitochondrial function (Molina et al., 2016; Zamani et al., 2021). The net result of these structural and biochemical alterations observed in HFpEF is a primary impairment in O_2_ diffusion (Houstis et al., 2018) and/or utilization resulting in depletion of high energy phosphates and early onset of skeletal muscle fatigue (Weiss et al., 2017).

## 10 EFFECTS OF EXERCISE TRAINING ON DETERMINANTS OF ${\dot{V}}_{O_{2}}$ IN HFpEF

Several investigations have been undertaken to examine the effect of supervised exercise training in patients with HFpEF. While the modality, frequency and duration of exercise interventions among these trials varies, supervised exercise training programmes are generally effective at improving functional capacity and quality of life. Indeed, meta‐analyses of randomized supervised exercise training in HFpEF show an increase relative peak ${\dot{V}}_{O_{2}}$ of ∼12% (2 ml/kg/min), a clinically meaningful improvement (Keteyian et al., 2010; Nayor et al., 2020; Sachdev et al., 2023). Regarding the mechanism of improvement in functional capacity, exercise training has been strikingly ineffective at improving left ventricular volume, pressure, compliance or function (stroke volume, cardiac output) during exercise (Fu et al., 2016; Fujimoto et al., 2012; Haykowsky et al., 2012; Mueller et al., 2021; Smart et al., 2012; Tucker et al., 2018). Therefore, it appears that non‐cardiac, peripheral adaptations are the primary mechanisms of improved peak ${\dot{V}}_{O_{2}}$ after exercise training in patients with HFpEF. Improvements in peak arterial‐venous oxygen content (a–vO_2_) difference are consistently improved by exercise training and can account for as much as 85% of the improvement in peak ${\dot{V}}_{O_{2}}$ (Borlaug et al., 2010; Fu et al., 2016; Fujimoto et al., 2012; Haykowsky et al., 2012; Tucker et al., 2018). However, the mechanisms responsible for improved peripheral oxygen utilization remain poorly understood. Exercise hyperaemia is determined primarily by the resistance vasculature of the skeletal muscle as opposed to the large elastic arteries typically assessed by flow mediated dilatation. Further, the mechanisms that govern exercise vasodilatation are likely more diverse that the mechanisms that govern traditional assessments of conduit vascular function (Hearon & Dinenno, 2016). Therefore, despite the limited ability of exercise training to improve these traditional markers of vascular function (Gevaert et al., 2023; Tucker et al., 2018), habitual exercise has been shown to improve vascular responses during acute bouts of exercise. A small investigation in patients with HFpEF who completed 8 weeks of isolated knee extensor exercise training showed improvements in leg vascular conductance (vasodilatation) during exercise compared to pre‐training values that was associated with an improvement in peak a–vO_2_ difference and functional capacity (Hearon, Samels et al., 2022). Therefore, some level of vascular plasticity may exist in patients with HFpEF. Less is known regarding the biochemical adaptation of skeletal muscle to exercise training. Findings from a large (*n* = 100) 20‐week intervention comparing caloric restriction to aerobic exercise training demonstrated that although neither intervention independently reduced intramuscular fat deposition, the change in the ratio of skeletal muscle to intermuscular fat was associated with improvements in peak ${\dot{V}}_{O_{2}}$ in patients with HFpEF (Kitzman et al., 2016). However, the combination of aerobic exercise and/or resistance exercise with caloric restriction was effective in lowering intramuscular fat deposition (Brubaker et al., 2022). Whether these markers of skeletal muscle metabolic health are accompanied by improvements in diffusive or oxidative capacity of skeletal muscle remains to be determined.

## 11 CLINICAL PHENOTYPES OF HFpEF

There is an increasing recognition that HFpEF is not a well‐defined clinical entity, such that patients with HFpEF may present with a combination of cardiovascular, metabolic, renal and/or geriatric conditions (Samson et al., 2016). The heterogeneity in clinical phenotypes of HFpEF contributes to the complex pathophysiology and manifestations that add great challenges to clinical care and warrants increased efforts to incorporate ‘phenomapping’ of this clinical syndrome (Cohen et al., 2020; Kao et al., 2015; Shah et al., 2015). This is important, because patients with HFpEF have a wide range of clinical profiles, including older age, female sex, history of hypertension, diabetes, obesity, atrial fibrillation, chronic kidney disease and coronary artery disease, and present with variable underlying cardiac and non‐cardiac abnormalities in structure and function (Lee et al., 2009; Owan et al., 2006; Shah & Pfeffer, 2012). Therefore, understanding how various clinical phenotypes of HFpEF differentially affect underlying pathophysiological processes will aid in identification of, and responsiveness to, targeted therapeutic interventions in this ever‐growing patient population (Peters et al., 2023).

## 12 SUMMARY

Exercise intolerance is a hallmark characteristic of patients with HFpEF that is likely the consequence of dysregulation across a spectrum of disease‐related changes in cardiovascular health, including deficits in cardiac function, increased arterial stiffness, peripheral vascular dysfunction, and impairments in oxygen delivery and utilization (Figure 1). Fortunately, there is now promising evidence for ‘plasticity’, particularly in the peripheral circulation, providing a renewed sense of optimism for the potential benefit of lifestyle and pharmacological therapies in this patient group.

## AUTHOR CONTRIBUTIONS

All authors conceived and discussed the content of the manuscript, approved the final version of the manuscript and agree to be accountable for all aspects of the work in ensuring that questions related to the accuracy or integrity of any part of the work are appropriately investigated and resolved. All persons designated as authors qualify for authorship, and all those who qualify for authorship are listed.

## CONFLICT OF INTEREST

The authors declare no conflicts of interest.
